# Investigating the factors that influence Chinese undergraduate students’ sustained use of open source communities

**DOI:** 10.1371/journal.pone.0308005

**Published:** 2024-12-27

**Authors:** Xinyi Wang, Rafiza Abdul Razak, Siti Hajar Halili

**Affiliations:** Department of Curriculum & Instructional Technology, Faculty of Education, Universiti Malaya, Kuala Lumpur, Malaysia; Beijing University of Technology, CHINA

## Abstract

The progress of open source technology is inseparable from cultivating open source talents in universities. The combination of open source communities and university education can cultivate students’ practical innovation abilities. Currently, we are facing problems such as the shortage of open source talents and the sustained use of open source technologies by open source talents. In addition, there are relatively few studies that explore the influencing factors of open source talents’ sustained use of open source communities, and research in this area plays a key role. This study aims to analyze the factors that influence the sustained use of open source communities among Chinese university students. The study used random stratified sampling to survey 803 undergraduate students in Yunnan Province. The influencing factors in innovation diffusion theory and the technology acceptance model were analyzed using the partial least squares structural equation model. Positive and significant effects among the seven factors of relative advantage, compatibility, trialability, observability, perceived ease of use, perceived usefulness, and perceived attitude, as well as the two model interrelationships, were demonstrated. In addition, perceived attitude and perceived usefulness have a significant positive impact on students’ sustained intention, and their mediating role is confirmed. In addition, the model is not affected by different students (gender, major, university, grade). This study provides valuable insights into the development of open source talent and the application of open source communities in education.

## Introduction

Open source will be incorporated into China as a national strategy to achieve new breakthroughs in the innovative application of key digital technologies, with the determination to move from an "open source country" to an "open source powerhouse" [1]. At the same time, society has a huge demand for open source talent, and there is a serious shortage of open source talent [2]. According to the major needs of China’s development, targeted training of open source talents [3]. Open source talent training is an urgent issue [4]. It can be seen that the cultivation of open source talents has an important impact on the development of China’s open source technology. Understanding open source talent’s acceptance of open source technology platforms can lead to better talent development. Currently, there are few studies on the willingness of open source talents in undergraduate schools to sustain using open source communities, especially in the context of China. To fill the research gap, it was learned that people believe that it is necessary to guide undergraduate universities to reform the curriculum system around the public welfare open source ecological environment and its infrastructure construction, and strengthen the training of open source software talents [5]. Researchers believe that the learning and practice of open source software development in teaching is an important way to enhance open source capabilities [5]. However, integrating the open source community into teaching can better exercise students’ open source ability. Some researchers argue that educational organizations should define open source software strategies in order to disseminate the principles and benefits of open source software taking into account the different needs of stakeholders (mainly students) and teachers when using open source communities for teaching [6]. As the researcher pointed out, "In the absence of sufficient external support and services, for institutional users, the user’s technical ability is a crucial factor affecting whether they are willing or able to use open source software" [7]. Some scholars believe that the depth and breadth of open source applications in Chinese universities are not enough, and they do not realize the importance of using open source [8]. Students lack the requisite technical knowledge and software experience for utilizing open source communities effectively. These problems prevent students from contributing to the open source community, accepting open source technology and sustaining to use of the open source community.

Based on these questions, we can see that when undergraduate students learn in the open source community, their sustained use intention affects their practice of open source technology. Although it is important to understand undergraduate students’ sustained intentions, there is little research discussion on undergraduate students’ sustained willingness to use open source communities in China, especially in related educational fields. Even if there are some studies, that mainly focus on the open source software of the bottom class, but there are very few studies on the open source software of the application class [9]. Also, few studies are discussed using mixed models. Therefore, there is an urgent need to study the factors that influence undergraduate students’ willingness to sustain using open source communities. Analyzing and understanding these factors is very important to promote the practical innovation ability of undergraduate students and cultivate high-quality open source talents. This study is proposed to fill the gap. From the perspective of Chinese undergraduates, this study comprehensively analyzes the factors that influence undergraduate students willingness to sustain using open source communities.

In summary, this study aims to analyze the factors influencing the sustained usage of open source communities among Chinese undergraduate students. These influencing factors are very important to educators and users and deserve further research. This study is proposed to fill in the gap; from the perspective of Chinese undergraduate students, a comprehensive analysis has been made on the influencing factors of Chinese undergraduate students’ willingness to sustain using open source communities in their learning. According to the research purpose of this study, we mainly answer the following four questions.

1. What is the relationship between Chinese undergraduate students’ IDT and TAM based on their willingness to sustain using the open source community?
2. What is the relationship between open source community based IDT-TAM and students’ intention of sustained usage (SISU) for Chinese undergraduate students?
3. What is the relationship between perceived attitude (PA) and perceived usefulness (PU) in Chinese undergraduate students sustaining the use intention TAM model based on open source community learning?
4. Are there significant differences in different demographic information levels, namely gender, major, university, and grade in TAM, IDT, and SISU based on the sustained use intention of Chinese undergraduate students in the open source community?

## Literature review

### Open source community

The open source community generally refers to software source code as the core, and developers who are geographically dispersed, but share common interests and hobbies, carry out knowledge creation such as joint development, maintenance, and enhancement of software in a democratic and cooperative form in accordance with the corresponding open source software license agreement. It is a network platform for communication and dissemination activities, and it is also a network organization for members to exchange learning and jointly manage [10]. The world’s largest open source website is GitHub, and Gitee is the largest and best open source website in China. It provides code hosting services based on Git. The Gitee has a community edition, enterprise edition, and university edition [11]. Some scholars study the factors that influence users’ knowledge contribution, thereby helping to formulate targeted strategies to promote user contribution behavior [12]. Open source communities are used in universities in China. For example, in schools in Sichuan areas, researchers have studied the information teaching of open source communities to train students’ ability to use Git warehouses [11]. In the Nanjing area, scholars use open source community virtual teaching to teach robot learning practice courses [13]. However, no relevant literature was found for the Yunnan region, which is a research gap that needs to be filled.

### IDT and TAM model

The fundamental theoretical foundation for this study is drawn from IDT and TAM. The Diffusion of Innovation Theory was proposed by American scholar E. M. Rogers in 1962 [14]. IDT refers to "the process by which an innovation spreads through certain channels among members of a social system over time" [15]. The key factors encompass relative advantage (RA), compatibility (CP), complexity, trialability (TL), and observability (OS) [15]. Davis proposed TAM in 1989 [16]. There are two influencing factors in the technology acceptance model, namely perceived ease of use and perceived usefulness, these two factors can affect users’ acceptance of technology [16]. Composed of these two determinants, the user’s attitude toward using technology determines the intention to use, which can determine the user’s actual usage behavior [16]. It’s easy to see, that IDT mainly analyzes people’s dissemination of new things, and TAM mainly analyzes users’ acceptance of technology. In addition, according to the literature review, it was found that IDT and TAM have been integrated by some researchers. Al-Rahmi et al. (2019), Shiau et al. (2018) and Almaiah et al. (2022) use IDT-TAM to analyze the factors that influence students and users’ sustained willingness to use technology is well validated, which is in line with the research purpose of this paper [17–19]. Since the IDT-TAM model has been confirmed by many researchers to be very suitable for studying users’ sustained use intentions, these two theoretical frameworks are very suitable for deriving factors that affect Chinese undergraduate students’ sustained use intentions for open source communities. In addition, The novelty of the model in this study is based on the IDT-TAM model, the researcher replaced the complexity of the IDT model with PE [20], and added control variables (gender, major, university, grade) to the original model.

Although IDT-TAM has been integrated by researchers, its use by Chinese undergraduate students in the open source community is almost nonexistent. In addition, the process of cultivating high-quality open source talents is an arduous task, so when cultivating talents, great efforts need to be made to influence the sustains use of open source communities by undergraduate students. Therefore, existing theories must be used as the research framework of this study to further promote a comprehensive and comprehensive understanding of undergraduate students’ willingness to sustain using open source communities. To do this, IDT and TAM models can be combined to measure relevant characteristics, including relative advantage, compatibility, trialability, observability, perceived ease of use, perceived usefulness, and perceived attitude. Next, based on academic inquiry, how these characteristics influence undergraduate students’ sustained use of open source communities will be discussed.

### Relative advantage of the IDT model

**Fig 1:**
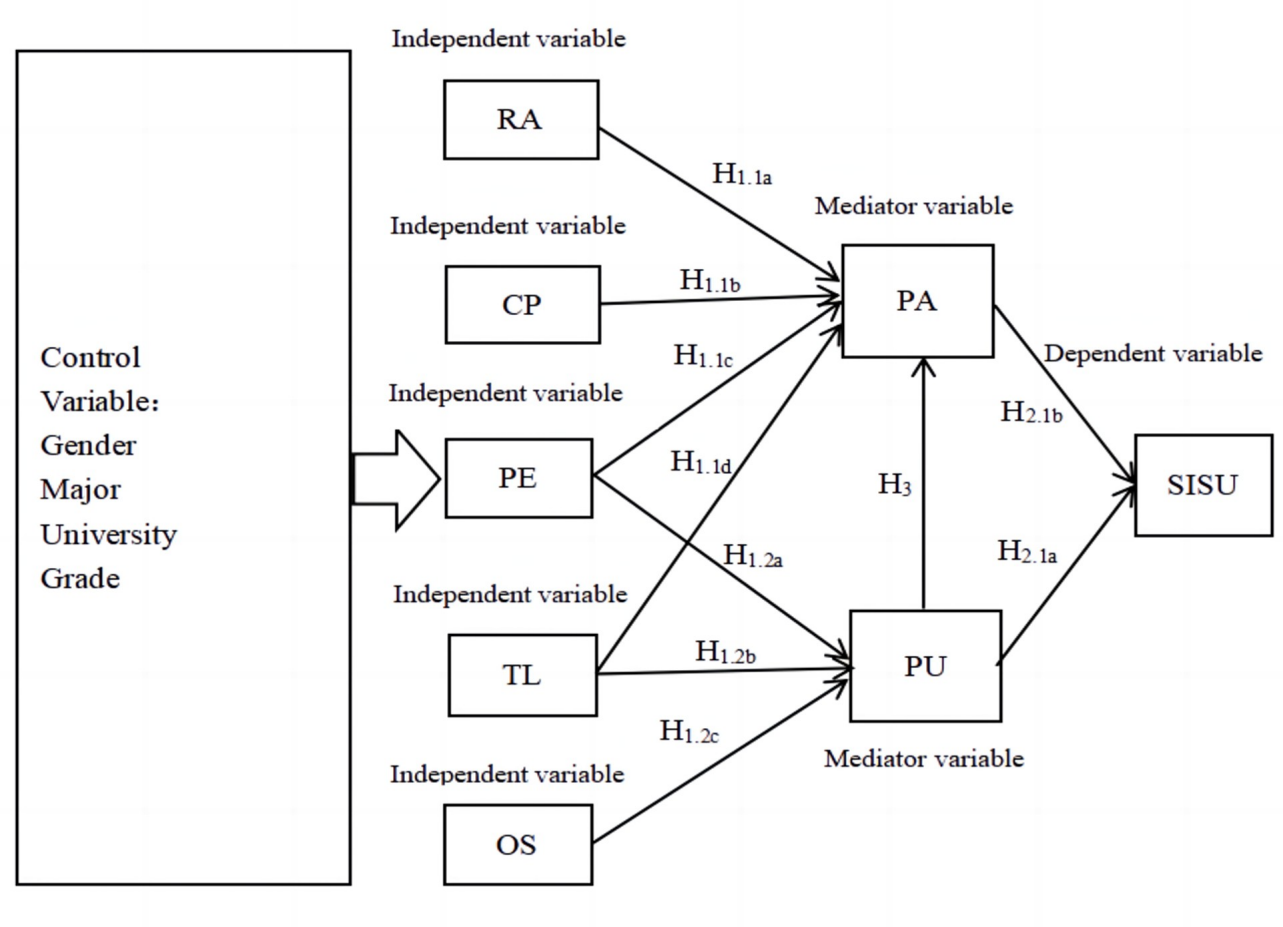
Conceptual framework.

Relative advantage (RA) refers to the extent to which individuals perceive a novel and innovative concept as superior to an old and conventional one. Relative advantage (RA) can be associated with various dimensions of learning platforms, including economic, social, convenience, and satisfaction aspects [21]. The relative advantage in this study refers to the advantages that undergraduate students perceive in learning in an open source community. In the current study, some researchers have surfaced the impact of relative advantage on users’ sustained use intention. As a technology, MOOC’s relative advantages have an impact on university students’ willingness to use MOOCs [22]. Relative advantage has an impact on tourists’ willingness to sustain using [23]. Relative advantage has an impact on academic users’ willingness to sustain using mobile visual search [24]. According to the research of these authors, it can be concluded that RA can have an impact on users’ sustained use intention. Besides, some researchers found that perceived attitude was significant as a mediator between relative advantage and sustained use intention [25, 26]. RA-PA-SISU as shown in Fig 1. We can see that perceived attitude plays an important role in terms of relative advantage and sustained use intention. Based on the above theory, the research hypothesis is proposed:

H_1.1a_: Relative advantage has a direct positive impact on perceived attitudes among undergraduate students learning in open source communities.

### Compatibility of IDT model

Compatibility (CP) primarily refers to the degree to which there is consistency between the innovation and the existing values, needs, and experiences of potential adopters [27]. This study uses the term to refer to undergraduate students’ perceptions of the benefits of using open source communities and their compatibility with the students’ own prior experiences. Compatibility is a key factor affecting the further proliferation of open and shared platforms for scientific and technological resources [28]. Some researchers found compatibility has a significant impact on users’ sustained use intention [29, 30]. Besides, according to the report of Wang et al. (2018), compatibility indirectly affects consumers’ adoption intentions through attitudes [31]. As shown in Fig 1 CP-PA-SISU. The research hypothesis is thus put forward:

H_1.1b_: Compatibility has a direct positive impact on perceived attitudes among undergraduate students learning in open source communities.

### Trialability of the IDT model

Trialability (TL) refers to the degree to which people think they need to experience the innovation before deciding whether to adopt it [32]. Trialability in this study refers to undergraduate students’ trial use of open source communities. Some studies [30, 33], in their study, discussed the relationship between sustained use intention and trialability. These studies found that intention to use sustained use was significantly influenced by trialability. However, in the study of Wang et al. (2018), it was found that trialability indirectly affects consumers’ adoption intention through attitude [31]. TL-PA-SISU as shown in Fig 1. Besides, in the study of Ullah et al. (2021), it was found that trialability was positively correlated with perceived usefulness [34]. As shown in Fig 1 TL-PU-SISU. The research hypothesis is:

H_1.1d_: Trialability has a direct positive impact on perceived attitudes among undergraduate students learning in open source communities.
H_1.2b_: Trialability has a direct positive impact on perceived usefulness among undergraduate students learning in open source communities.

### Observability of the IDT model

Observability (OS) is defined as the extent to which an innovation is noticed by others. Rogers et al. (2010) pointed out that OS refers to the degree to which an innovation is visible to others [15]. Based on this perspective, the observability of this study means that undergraduate students can observe other people’s usage when using the open source community. Through studies on different populations, it has been found that observability can well predict the willingness to sustain using [17, 35]. Among them, studies by Bharadwaj & Deka (2021) and Al-Rahmi et al. (2019) show that observability is positively correlated with perceived usefulness [17, 35]. As shown in Fig 1 OS-PU-SISU. The research hypothesis is thus put forward:

H_1.2c_: Observability has a direct positive impact on perceived usefulness among undergraduate students learning in open source communities.

### Demographic aspects of the IDT model

In innovation diffusion theory (IDT), complexity is replaced by Perceived ease of use. Based on the theory supporting the questionnaire design, the perceived ease of use dimension takes precedence over the complexity determinant in the survey [36]. Some researchers have explored the lack of impact of gender on sustained knowledge contributions in online communities [37]. Furthermore, perceptions about innovation diffusion theory and students’ sustained use are influenced by a variety of factors, including but not limited to age, gender, and university. Users of different genders and ages have differences in user adoption behavior [38, 39]. In the research of Du (2019), it was found that there are significant differences among students of different types of colleges and universities [22].

### Perceived ease of use of the TAM model

Perceived ease of use is defined as the degree to which a particular system can be used without much effort on the part of the user [16]. Perceived ease of use in this study refers to the extent to which undergraduate students believe that the open source community is easy to use and learn from. Some scholars believe that perceived ease of use can well predict perceived attitude, and thus indirectly predict users’ sustained use intention through perceived attitude. [40–43]. As shown in PE-PA-SISU in Fig 1. In addition, scholars found that PE using the metadata learning platform in universities cannot directly affect behavioral intentions, but PU can [44]. Some scholars believe that perceived ease of use can also indirectly affect users’ sustained use willingness through perceived usefulness [40, 42, 45]. PE-PU-SISU as shown in Fig 1. Based on the above theory, the research hypothesis is proposed:

H_1.1c_: Perceived ease of use has a direct positive impact on perceived attitudes among undergraduate students learning in open source communities.
H_1.2a_: Perceived ease of use has a direct positive impact on perceived usefulness among undergraduate students studying in open source communities.

### Perceived usefulness of the TAM model

The extent to which users believe that using a particular system can contribute to the user’s job performance is the definition of perceived usefulness [16]. This study used perceived usefulness, which is the extent to which undergraduate students believe that the open source community is useful for their studies. In the research of some scholars, it is found that perceived usefulness can directly predict a user’s sustained use intention [40, 46, 47]. PU-SISU as shown in Fig 1. At the same time, regarding the fact that perceived usefulness can indirectly affect users’ willingness to sustain using through perceived attitude, some scholars’ studies have confirmed this point [40–43]. PU-PA-SISU as shown in Fig 1. The research hypothesis is thus put forward:

H_3_: Perceived usefulness has a direct positive impact on perceived attitude among undergraduate students in open source community learning.
H_2.1a_: For undergraduate students learning in an open source community, perceived usefulness has a direct positive impact on student’s intention of sustained usage.

### Perceived attitude of the TAM model

Perceived attitude (PA) is a physiological inclination expressed through the evaluation of a specific entity, indicating a level of preference or aversion [16]. Perceived attitudes in the study refer to undergraduate students’ attitudes toward the open source community. Perceived attitudes directly influence technology usage intentions [40, 41, 48]. As shown in Fig 1 PA-SISU. The research hypothesis is:

H_2.1b_: Undergraduate students’ perceived attitude has a direct positive impact on student’s intention of sustained usage when learning in an open source community.

### Demographic aspects of the TAM model

In the Technology Acceptance Model (TAM), researchers looked at students’ attitudes towards and use of Information and Communication Technology (ICT) and found differences in university type factors [49]. Other researchers have found that seniors show a higher behavioral willingness to learn through e-learning, and first-, second- and third-year students reject e-learning [50]. In Wang and Houdyshell (2021) study, it was found that students had no significant difference in knowledge or experience of Remote Academic Advising according to demographic independent variables of gender and major [51].

### Students’ intention of sustained usage (SISU)

Students’ intention to sustain use (SISU) in this study is described as Chinese undergraduate students’ intention to sustain the use of open source communities. SISU is affected by many factors [52]. For example, some researchers have pointed out that online interaction has an impact on the participants’ continuous knowledge contribution [53]. In addition, it is also pointed out that knowledge reciprocity has a positive impact on the participants’ continuous knowledge contribution [54]. Community recognition is one of the biggest factors affecting the participants’ knowledge contribution [55]. Social interaction has a moderating effect on users’ continuous knowledge contribution [56]. According to the previous discussion, we can find that perceived usefulness has a positive effect on students’ sustain of intention to use. Similarly, perceived attitude also has a positive effect on students’ sustained intention to use.

The mixed model of IDT-TAM means that the structure of TAM becomes part of IDT; compared with using either of them alone, the two models are mixed to obtain a more effective framework [57–59]. From the structures of IDT and TAM, there are obvious complementarities and similarities between them [18]. According to the literature review, scholars use the IDT-TAM model for analysis when studying users’ sustained use intention, and the research results show that the IDT-TAM model can well predict users’ sustained use intention [17, 18, 60].

Through the review of the above literature, it is found that a large number of research surveys have provided empirical evidence to support the analysis of factors affecting undergraduate students’ acceptance and sustained use of open source technology, which is a prerequisite for cultivating open source talents. Therefore, using the IDT-TAM mixed model can well identify factors that influence undergraduate students’ willingness to sustain using open source communities. Furthermore, exploring how undergraduate students’ gender, major, university, and grade affect their use of open source communities is an area that has been barely researched. In Fig 1, the researcher reports the conceptual framework of this study.

## Research method

### Research design

This study employs a statistical technique called Partial Least Squares Structural Equation Modeling (PLS-SEM) to develop a framework that combines IDT theory and TAM models. The purpose is to predict and elucidate the factors influencing Chinese undergraduate students’ willingness to sustain using open source communities. This framework examines the correlation between students’ sustained willingness to use and seven key variables: RA, CP, TL, OS, PE, PU, PA, and SISU.

### Sampling and data collection

This study selected undergraduate students in Yunnan Province, China as a sample, because this study examined undergraduate students’ sustained use of open source technologies in their learning in an open source community. There are 32 undergraduate universities in Yunnan Province with a large number of undergraduate students, and more than 2,500 universities in China have cooperated with open source communities. Therefore, the research on the willingness of Yunnan undergraduate students to sustain to use of open source communities can be considered representative. The sampling technique employed was "stratified" random sampling. The researchers contacted teachers from different undergraduate universities in Yunnan Province whom they were acquainted with, and the researcher then made an appointment with these teachers, and the researcher arrived at the site and sent the data to the undergraduate students to fill out. The investigation period begins on September 25, 2023, and ends on October 25, 2023. The researchers used the online platform to distribute 900 questionnaires to undergraduate students, and collected 840 valid answers, after deleting outliers and missing values, the data is 803. Such a sample size can be considered representative of the overall population and can well ensure the reliability of the study [61]. In the questionnaire survey, participants can review the informed consent form at any time and indicate their own information by checking the checkboxes. The participants in this study are all undergraduate students over the age of 18. The questionnaire survey time of this study was 1 month. Among them, demographic information includes gender, age, major, university, and grade. During the receipt collection process, the researchers strictly abide by the research ethics standards, and keep the information provided by the participants strictly confidential and safe.

The samples are valid questionnaires collected by the online platform. In data preparation, the researcher cleans the data to remove outliers and missing values. The final valid data set is 803. The gender distribution is that males account for 67.9% of the total number of participants and females account for 32.1% of the total number of participants. The above gender differences are caused by the different ratios of male and female students in the majors studied. Such results have a certain degree of universality. Moreover, according to the results of the subsequent one-way ANOVA, the research results are not affected by gender diversity. In Table 9. 33.4% of undergraduate students are 18–19 years old, 32.4% are 20–21 years old, and 34.2% are older than 22 years old. Private universities and public universities accounted for 53.4% and 46.6% respectively. Computer Science and Technology (CSAT) majors accounted for 44.6%, Electronic Information Science and Technology (EISAT) majors accounted for 21.9%, Software Engineering (SE) majors accounted for 20.9%, and Electrical Information (EI) majors accounted for 12.6%. 1–2 semesters accounted for 24.3%, 3–4 semesters accounted for 28.1%, 5–6 semesters accounted for 24.7%, and 7–8 semesters accounted for 22.9% in Table 1.

**Table 1:** Respondents’ gender, age, grade, major, and university based on open source community.

No. 803	Classification	Frequency	Percentage (%)
Gender	Male	545	67.9
	Female	258	32.1
Age	18–19	268	33.4
	20–21	260	32.4
	>22	275	34.2
University	Private university	429	53.4
	Public university	374	46.6
Major	Computer Science and Technology(CSAT)	358	44.6
	Electronic Information Science and Technology (EISAT)	176	21.9
	Software Engineering (SE)	168	20.9
	Electrical Information (EI)	101	12.6
Grade	1–2 semesters	195	24.3
	3–4 semesters	226	28.1
	5–6 semesters	198	24.7
	7–8 semesters	184	22.9

### Research tool

Researchers revise and predict questionnaires to collect data on key factors needed for undergraduate students’ sustained intention to use open source communities. The questionnaire comprises components of the proposed conceptual model, including RA, CP, TL, OS, PE, PA, PU, and SISU. The research questionnaire uses Likert’s 5-point scale to effectively measure the influencing factors. The answers to the questionnaire are mainly made in a multiple-choice manner, with answers ranging from "strongly disagree" to "strongly agree". The design of the research questionnaire was based on a review of the literature and adaptation of research scale items from previous studies. The purpose of this is to ensure the relevance and appropriateness of the questionnaire. All items are reliable in Table 2.

**Table 2:** Questionnaire structure and indicators.

Construct	No of Indicators	Indicators	Adopted and modified from	Cronbach’s alpha
Relative Advantage (RA)	6	RA1, RA2, RA3, RA4, RA5, RA6	Stark(2018)	0.864
Compatibility (CP)	6	CP1, CP2, CP3, CP4, CP5, CP6	Meng(2021)	0.868
Trialability (TL)	5	TL1, TL2, TL3, TL4, TL5	Dotter(2018)	0.844
Observability (OS)	3	OS1, OS2, OS3	Pinho et al., (2021)	0.773
Perceived Usefulness (PU)	5	PU1, PU2, PU3, PU4, PU5	Zhang et al., (2015)	0.851
Perceived ease of Use (PE)	5	PE1, PE2, PE3, PE4, PE5	Zhang et al., (2015)	0.845
Perceived Attitude (PA)	5	PA1, PA2, PA3, PA4, PA5	Ab Jalil et al., (2019)	0.844
The student’s intention of sustained usage(SISU)	3	SISU1, SISU2, SISU3	Li(2016)	0.798

### Results

The research mainly uses bootstrapping and the Smart PLS 4 algorithm. This approach can support the modeling of complex relationships covering many indicator variables and paths [61]. This study implements the predictive method of model estimation, so PLS-SEM is the preferred software for data analysis of the study. Because one of the main functions of PLS-SEM is to deal with the dichotomy between information, previous relevant theories, and prediction as to the development foundation [61]. Furthermore, PLS-SEM is the most suitable research method for exploring or extending existing structural theories [61]. The researcher will answer research questions 1–3.

### Measurement model

At this stage, we first considerations are validity and reliability indicators. Test results for reliability are reported in Table 3. The purpose of this reliability test is to determine the consistency among the items of each facet. Cronbach’s Alpha value is an indicator to detect the internal consistency of the model, see Table 3. At the same time, the researchers also assessed convergent and discriminant validity among the facets. The test results are shown in Tables 4 and 5.

**Table 3:** Measure model results.

Constructs	Indicators	Loading	α	CR	AVE
Compatibility	CP1	0.784	0.870	0.902	0.605
	CP2	0.788			
	CP3	0.779			
	CP4	0.750			
	CP5	0.776			
	CP6	0.790			
Observability	OS1	0.835	0.783	0.874	0.697
	OS2	0.846			
	OS3	0.824			
Perceived Attitude	PA1	0.778	0.852	0.894	0.628
	PA2	0.808			
	PA3	0.793			
	PA4	0.783			
	PA5	0.801			
Perceived Ease of Use	PE1	0.785	0.845	0.889	0.617
	PE2	0.805			
	PE3	0.791			
	PE4	0.787			
	PE5	0.759			
Perceived Usefulness	PU1	0.781	0.842	0.888	0.614
	PU2	0.754			
	PU3	0.809			
	PU4	0.794			
	PU5	0.777			
Relative Advantage	RA1	0.779	0.869	0.902	0.605
	RA2	0.736			
	RA3	0.792			
	RA4	0.802			
	RA5	0.770			
	RA6	0.786			
The student’s intention of sustained usage	SISU1	0.836	0.794	0.879	0.708
	SISU2	0.840			
	SISU3	0.848			
Trialability	TL1	0.794	0.847	0.891	0.621
	TL2	0.800			
	TL3	0.764			
	TL4	0.809			
	TL5	0.771			

**Table 4:** Measurement model discriminant validity.

	CP	OS	PA	PE	PU	RA	SISU	TL
CP	0.778							
OS	0.542	0.835						
PA	0.561	0.547	0.793					
PE	0.547	0.532	0.564	0.785				
PU	0.551	0.538	0.562	0.563	0.783			
RA	0.596	0.545	0.538	0.564	0.596	0.778		
SISU	0.556	0.529	0.545	0.575	0.558	0.555	0.842	
TL	0.570	0.575	0.548	0.546	0.573	0.595	0.552	0.788

**Table 5:** Heterotrait-monotrait ratio (HTMT) results.

	CP	OS	PA	PE	PU	RA	SISU
OS	0.656						
PA	0.651	0.669					
PE	0.638	0.654	0.665				
PU	0.643	0.661	0.662	0.666			
RA	0.685	0.660	0.623	0.657	0.695		
SISU	0.669	0.670	0.663	0.701	0.682	0.668	
TL	0.664	0.706	0.645	0.644	0.677	0.693	0.672

The research results show that Cronbach’s alpha values of all variables exceed the critical value of 0.6, so it can be considered that all potential endogenous variables are reliable. When the value is between 0.7 and 0.8, the value is satisfactory; when the value is between 0.7 and 0.9, the value is acceptable [61]. In addition, we can see that the loading values of all facets are greater than 0.700 in Table 3. So, proving the consistency of items in each facet.

We can see that the AVE (Average Variance Extraction) value exceeds 0.5 and the composite reliability of all facets exceeds 0.7 in Table 3 [61]. Hence, these facets exhibit convergent validity. The researchers used the Fornell and Larcker method and the heterotrait-monotropy ratio (HTMT) method to evaluate the discriminant validity of the model [61]. According to this method, the researchers calculated the square root of the Average Variance Extracted or the square root of each latent aspect through smart-pls and checked its correlation with various latent constructs. It should be emphasized that all diagonal values are larger than other related values, meeting the criteria for discriminant validity in Table 4. HTMT also fits the bill, in Table 5.

### Structural model

This part of the assessment consists of 5 steps. In the first step, multicollinearity was tested by evaluating the variance inflation factor (VIF) among the various constructs. The second step is to test the T value and P value. The third step is to calculate the R2 value to test the coefficient of determination. The fourth step is to calculate the f2 value to test the model effect size. Finally, the predictive power of the model is evaluated by determining the Q2 value. As all factors in the model are reflective, the researchers employed VIF for assessment. Presents the VIF values between predicted endogenous structures. As can be seen, all VIF values are much less than 5, indicating that the model does not have the problem of multicollinearity, which proves that the evaluation results of the model are satisfactory in Table 6 [61].

**Table 6:** Internal VIFs for predictors.

	PA	PU	SISU
CP	1.901		
OS		1.664	
PA			1.461
PE	1.818	1.586	
PU	1.932		1.461
RA	2.073		
SISU			
TL	1.933	1.699	

In addition, the researchers also used bootstrapping to test the structural model of this study. The researchers list R2, effect size (f2), and Q2 In Table 7. The researchers then used blindfolding to validate the model’s predictive potential. According to the report, the model in this study has a certain predictive ability, as all endogenous structures had Q2 values exceeding 0 in Table 7.

**Table 7:** R^2^, effect size(f^2^)and Q^2^.

	R^2^	f ^2^			Q^2^
		PA	PU	SISU	
CP		0.037			
OS			0.051		
PA	0.472			0.128	0.293
PE		0.047	0.095		
PU	0.446	0.035		0.152	0.270
RA		0.009			
SISU	0.390				0.273
TL		0.024	0.089		

**Fig 2:**
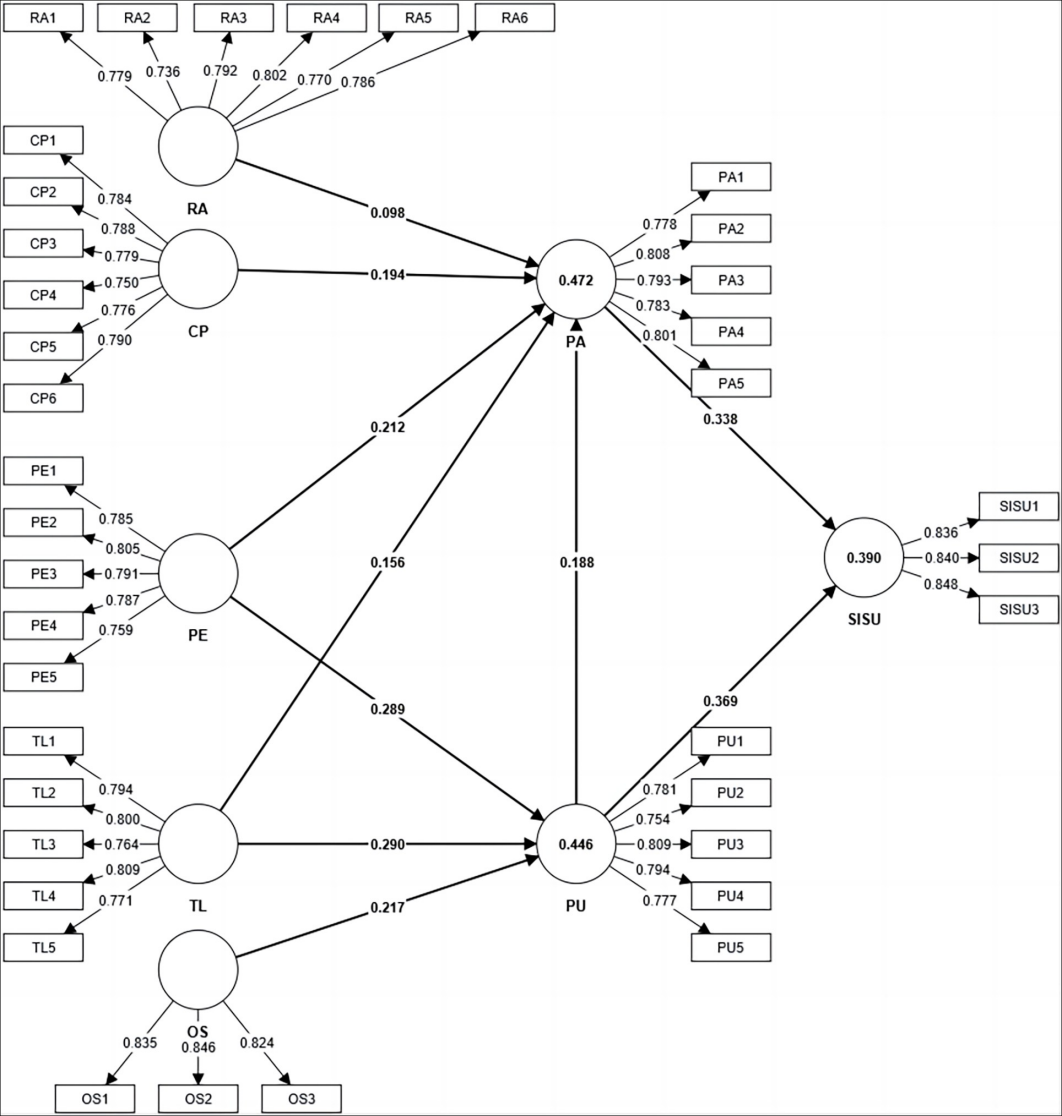
Based on the influencing factor model of undergraduate students’ willingness to sustain using open source communities.

We can see the path analysis results of the model in Fig 2 and Table 8. First, on the perceived attitude side. Relative advantage and perceived attitude *(RA -> PA; β = 0*.*098; p < 0*.*01)*, proving that RA and PA are positively significant, supporting hypothesis H_1.1a_. Compatibility and perceived attitude *(CP -> PA; β = 0*.*194; p < 0*.*01)*, CP has a significant positive relationship with PA. This result supports the research hypothesis of H_1.1b_. Trialability and perceived attitude *(TL -> PA; β = 0*.*156; p < 0*.*01)*. It is shown that there is a positive significance between TL and PA, and the research hypothesis H_1.1d_ is supported. Perceived ease of use and perceived attitude *(PE -> PA; β = 0*.*212; p < 0*.*01)*, indicating a positive significance between PE and PA, and the research hypothesis H_1.1c_ is supported. According to the above results, RA, CP, TL, and PE can directly and positively affect PA. In addition, compatibility, perceived ease of use, relative advantage, and trialability indirectly affect student’s intention of sustained usage through perceived attitude, with significant positive effects *(PE ->PA->SISU*, *CP->PA->SISU*, *TL->PA -> SISU; p < 0*.*01) (RA -> PA -> SISU; p < 0*.*05)*. In other words, PA plays a mediating role among CP, PE, RA, TL, and SISU and exerts a positive influence among them. In addition, perceived usefulness has a significant impact on perceived attitude *(PU -> PA; β = 0*.*188; p < 0*.*01)*, proving that PU and PA are positively significant, supporting hypothesis H_3_. This shows that students’ perceived attitudes can also be directly affected by PU. Furthermore, trialability, perceived ease of use, and observability significantly and positively influence perceived attitude through perceived usefulness *(TL -> PU -> PA*, *PE -> PU -> PA*, *OS -> PU -> PA; p<0*.*01)*. In other words, PU mediates the relationship between TL, PE, OS, and PA. In addition, perceived usefulness indirectly and positively affects student’s intention of sustained usage through perceived attitude *(PU -> PA -> SISU; p < 0*.*01)*. This indicates that PA mediates the relationship between PU and SISU.

**Table 8:** Path analysis results.

Path	Effect type	β	T-value	Significance
CP -> PA	Direct effect	0.194	5.454	Yes ***
PE -> PA	Direct effect	0.212	6.190	Yes ***
RA -> PA	Direct effect	0.098	2.703	Yes ***
TL -> PA	Direct effect	0.156	4.356	Yes ***
PE -> PA -> SISU	Indirect effect		4.999	Yes ***
CP -> PA -> SISU	Indirect effect		4.538	Yes ***
TL -> PA -> SISU	Indirect effect		3.823	Yes ***
RA -> PA -> SISU	Indirect effect		2.566	Yes **
PE -> PU	Direct effect	0.289	8.582	Yes ***
TL -> PU	Direct effect	0.290	8.457	Yes ***
OS -> PU	Direct effect	0.217	6.571	Yes ***
PE -> PU -> SISU	Indirect effect		6.452	Yes ***
TL -> PU -> SISU	Indirect effect		6.349	Yes ***
OS -> PU -> SISU	Indirect effect		5.537	Yes ***
PU -> PA	Direct effect	0.188	5.073	Yes ***
PU -> PA -> SISU	Indirect effect		4.784	Yes ***
PA -> SISU	Direct effect	0.338	10.060	Yes ***
PU -> SISU	Direct effect	0.369	10.835	Yes ***
TL -> PU -> PA	Indirect effect		4.511	Yes ***
PE -> PU -> PA	Indirect effect		4.435	Yes ***
OS -> PU -> PA	Indirect effect		3.621	Yes ***
TL -> PU -> PA -> SISU	Indirect effect		4.329	Yes ***
PE -> PU -> PA -> SISU	Indirect effect		4.280	Yes ***
OS -> PU -> PA -> SISU	Indirect effect		3.528	Yes ***

Note

***p < 0.01

**p < 0.05

*p < 0.10

Secondly, in terms of the impact on perceived usefulness. Trialability and perceived usefulness *(TL -> PU; β = 0*.*290; p < 0*.*01)*, TL and PU have a significant positive relationship. This result supports H_1.2b_ in this research hypothesis. Observability has a positive significance on perceived usefulness *(OS -> PU; β = 0*.*217; p < 0*.*01)*, indicating a positive significance between OS and PU, and the research hypothesis H_1.2c_ is supported. Perceived ease of use and perceived usefulness *(PE -> PU; β = 0*.*289; p < 0*.*01)*, prove that PE and PU are positively significant, supporting the hypothesis H_1.2a_. The research results show that PE, TL, and OS can directly and positively affect PU. In addition, perceived ease of use, trialability, and observability can also have an indirect and significant positive impact on student’s intention of sustained usage through perceived usefulness *(PE->PU->SISU*, *TL->PU->SISU*, *OS->PU->SISU; p < 0*.*01)*. It is confirmed that the intermediary nature of PU is that PE, TL, and OS have a positive impact on SISU under the intermediary effect of PU.

Finally, regarding student’s intention of sustained usage. Perceived attitude has a positive impact on student’s intention of sustained usage *(PA -> SISU; β = 0*.*338; p < 0*.*01)*, indicating a positive significance between PA and SISU. The study hypothesis H_2.1b_ is supported. Perceived usefulness has a positive impact on student’s intention of sustained usage *(PU -> SISU; β = 0*.*369; p < 0*.*01)* and has a significant impact. It is proved that PU and SISU are positively significant, supporting hypothesis H_2.1a_. The research results show that PA and PU can directly and positively affect students’ intention of sustained usage. In addition, trialability, perceived ease of use, and observability have significant positive effects on student’s intention of sustained usage through perceived usefulness and perceived attitude *(TL -> PU -> PA -> SISU*, *PE -> PU -> PA -> SISU*, *OS -> PU -> PA -> SISU; p < 0*.*01)*. From the above indirect path results, it can be seen that perceived attitude and perceived usefulness play a mediating role in the relationship between IDT and SISU.

The researchers tested the model’s predictive power, the R2 value. When R2 is 0.25 belongs to weak, 0.50 belongs to medium and 0.75 belongs to large [61]. Based on the findings, the effects of perceived attitude, perceived usefulness, and students’ willingness to sustain using are close to moderate in Table 7. In addition, the researchers examined the effect size (f2), which was assessed as 0.02 for a small, 0.15 for a medium, and 0.35 for a large. According to the finding, perceived usefulness (f2 = 0.152) has a moderate effect on students’ willingness to sustain using in Table 7. Relative advantage (f2 = 0.009) had little effect on perceived attitude. At the same time, the effect sizes of the remaining influencing factors are small. We can see that in terms of model predictive ability, the proposed model has predictive ability, because the Q2 values of all endogenous constructs calculated by the blindfold method are greater than 0 in Table 7. The Q2 value was evaluated as 0.02 with small foreign structures, 0.15 with medium foreign structures, and 0.35 with large foreign structures. So, the model has a moderate exogenous structure.

### Descriptive results

**Fig 3:**
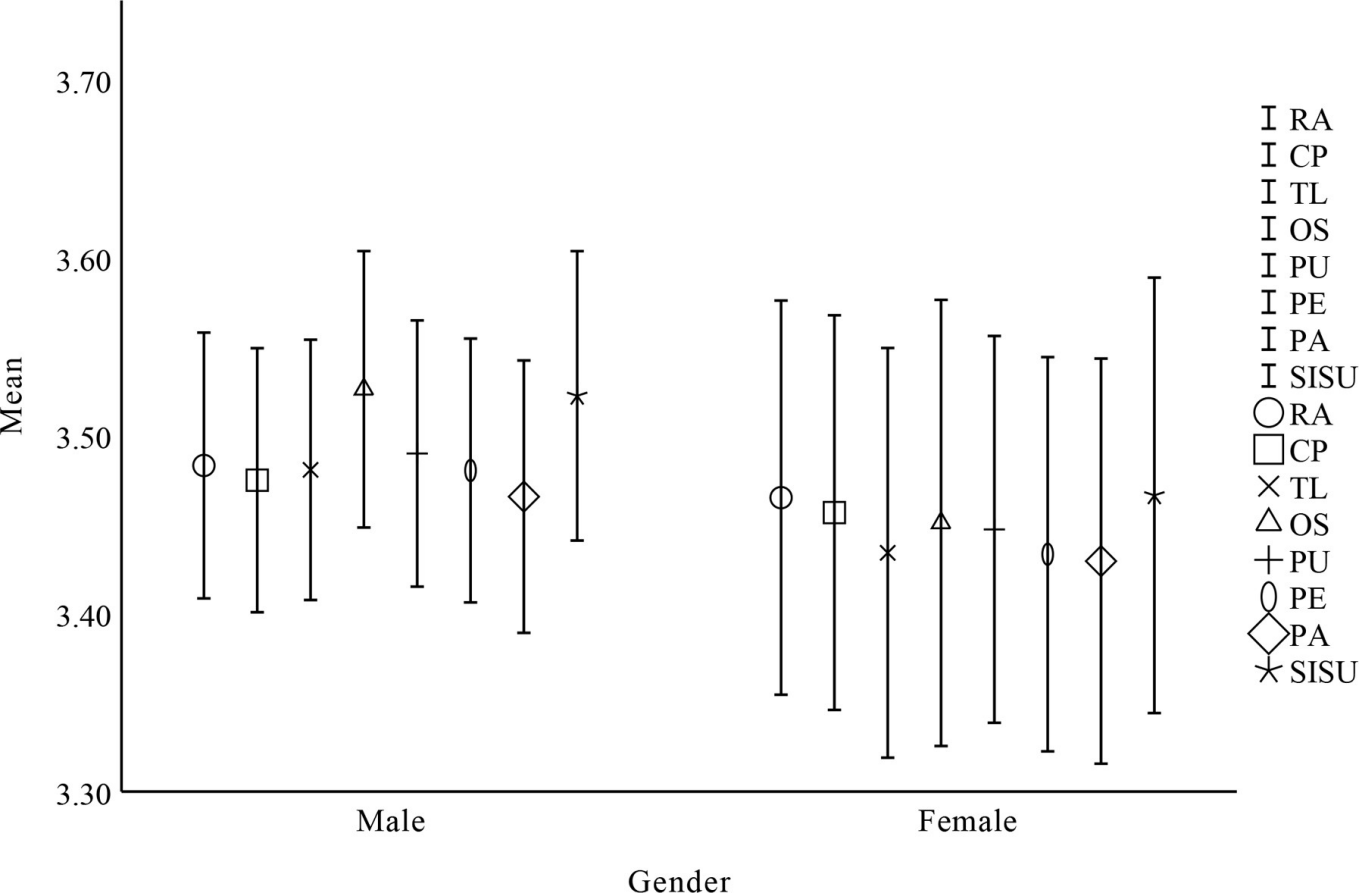
One-way ANOVA results for gender.

**Fig 4:**
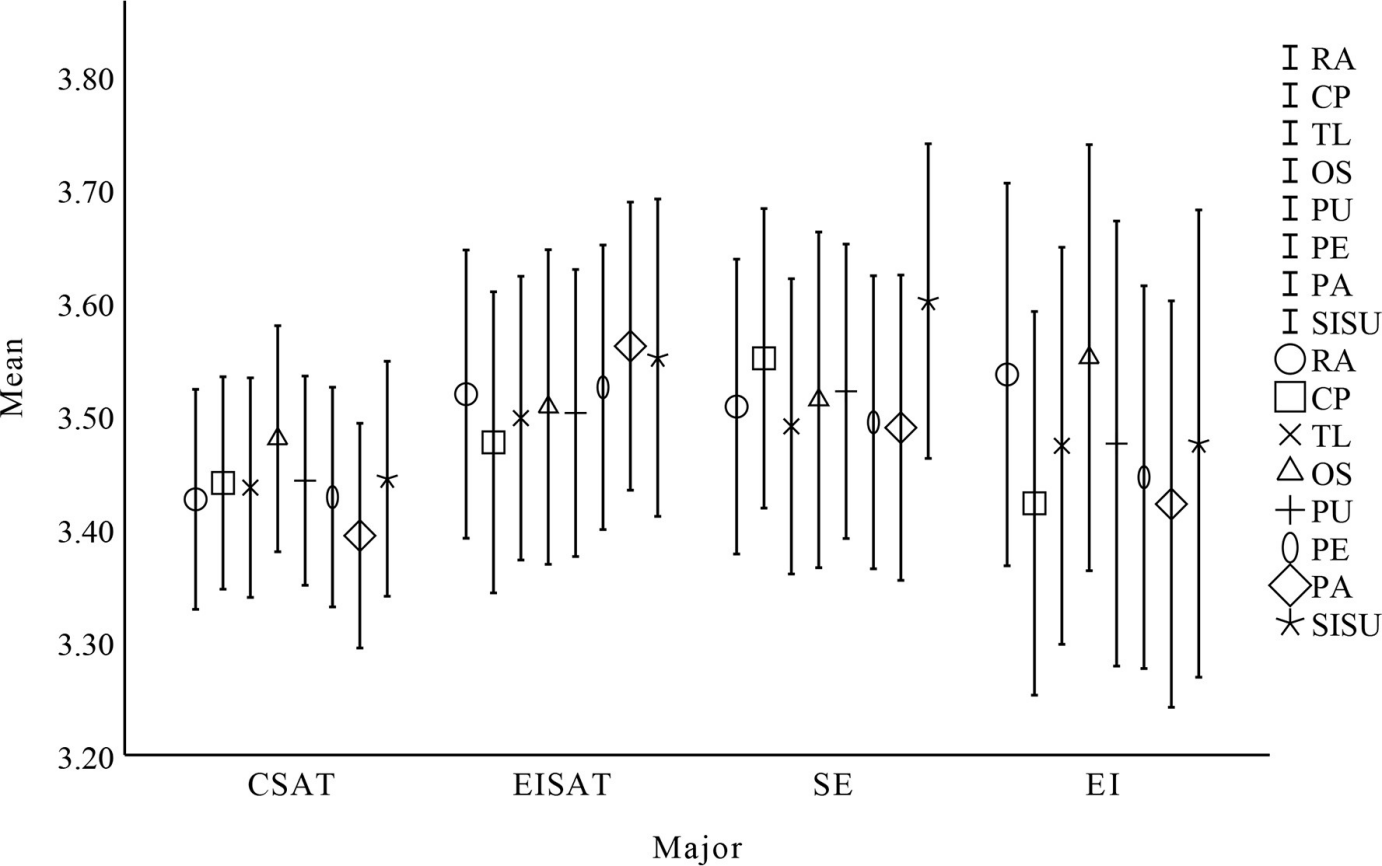
One-way ANOVA results for major.

**Fig 5:**
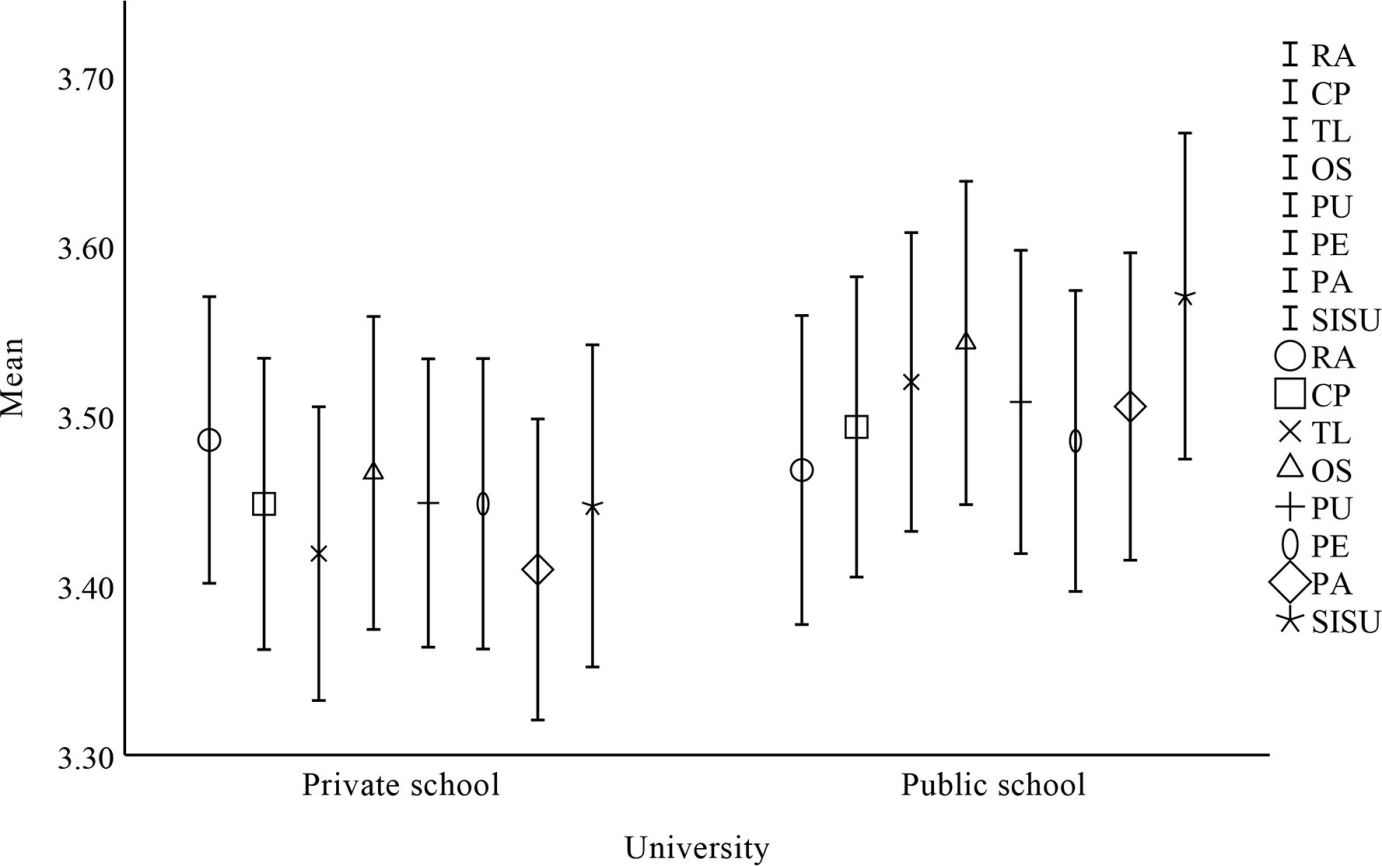
One-way ANOVA results for university.

**Fig 6:**
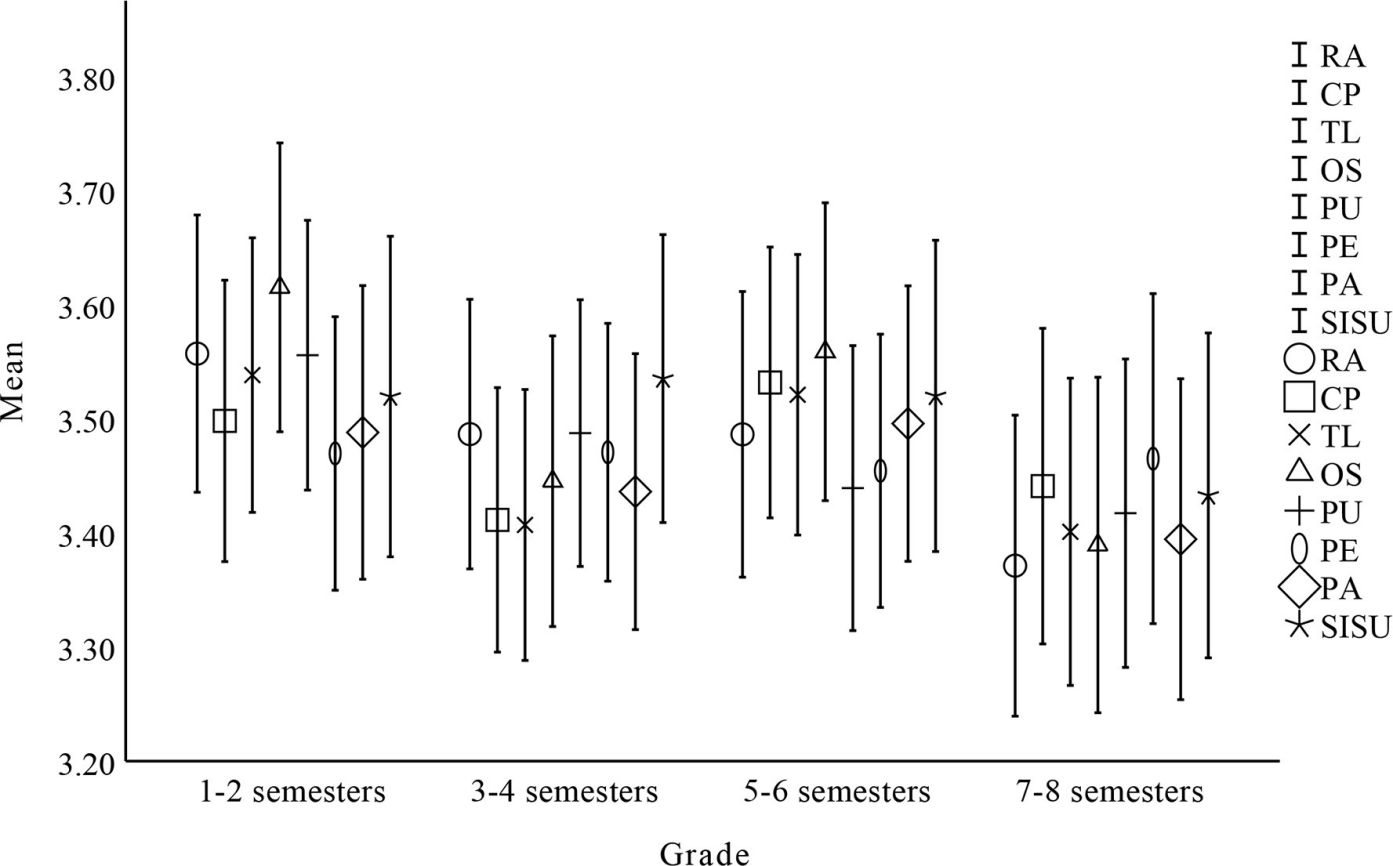
One-way ANOVA results for grade.

Answer research question 4 based on the reported results in Figs 3–6 and Tables 9–12. That is the influence of gender, major, university, and grade on the willingness of undergraduate students to sustain using open source communities. To research demographic differences (gender, major, university, and grade level) among students in their willingness to sustain using open source communities, the researchers used a one-way ANOVA. Since there are only 2 groups of gender and university, and major and grade show no significant correlation, there is no need for post hoc testing. Before conducting one-way ANOVA, researchers applied Levene’s test to evaluate variance homogeneity among cases. The findings showed that there were differences in some factors, so the researchers used Welch and Brown-Forsythe’s analysis results to evaluate. According to the report, in terms of gender, major, university, and grade, RA, CP, PE, TL, OS, PA, and PU did not significantly impact the willingness of undergraduate students to sustain using open source communities (P>0.05).

**Table 9:** Factors affecting undergraduate students’ sustain use intention based on gender.

Factor	Gender	N	Mean	SD	SE	df	F	P
RA	Male	545	3.483	0.887	0.038	1	0.072	0.790
	Female	258	3.465	0.903	0.056			
CP	Male	545	3.475	0.881	0.038	1	0.073	0.788
	Female	258	3.457	0.904	0.056			
TL	Male	545	3.481	0.869	0.037	1	0.479	0.501
	Female	258	3.434	0.939	0.058			
OS	Male	545	3.526	0.923	0.040	1	1.081	0.317
	Female	258	3.451	1.022	0.064			
PU	Male	545	3.490	0.889	0.038	1	0.404	0.525
	Female	258	3.447	0.886	0.055			
PE	Male	545	3.480	0.881	0.038	1	0.491	0.488
	Female	258	3.433	0.903	0.056			
PA	Male	545	3.466	0.909	0.039	1	0.274	0.603
	Female	258	3.430	0.928	0.058			
SISU	Male	545	3.522	0.966	0.041	1	0.574	0.454
	Female	258	3.466	0.998	0.062			

**Table 10:** Factors affecting undergraduate students’ sustain use intention by major.

Factor	Major	N	Mean	SD	SE	df	F	P
RA	CSAT	358	3.426	0.935	0.049	3	0.736	0.540
	EISAT	176	3.519	0.856	0.065			
	SE	168	3.508	0.855	0.066			
	EI	101	3.536	0.857	0.085			
CP	CSAT	358	3.440	0.903	0.048	3	0.692	0.545
	EISAT	176	3.476	0.895	0.067			
	SE	168	3.551	0.869	0.067			
	EI	101	3.422	0.859	0.085			
TL	CSAT	358	3.436	0.933	0.049	3	0.250	0.862
	EISAT	176	3.498	0.843	0.064			
	SE	168	3.491	0.857	0.066			
	EI	101	3.473	0.889	0.088			
OS	CSAT	358	3.480	0.961	0.051	3	0.165	0.920
	EISAT	176	3.508	0.935	0.070			
	SE	168	3.514	0.974	0.075			
	EI	101	3.551	0.954	0.095			
PU	CSAT	358	3.443	0.890	0.047	3	0.368	0.767
	EISAT	176	3.502	0.853	0.064			
	SE	168	3.521	0.854	0.066			
	EI	101	3.475	0.996	0.099			
PE	CSAT	358	3.428	0.934	0.049	3	0.551	0.642
	EISAT	176	3.525	0.845	0.064			
	SE	168	3.494	0.850	0.066			
	EI	101	3.446	0.857	0.085			
PA	CSAT	358	3.394	0.956	0.051	3	1.450	0.212
	EISAT	176	3.561	0.856	0.065			
	SE	168	3.489	0.886	0.068			
	EI	101	3.422	0.910	0.091			
SISU	CSAT	358	3.444	0.999	0.053	3	1.171	0.302
	EISAT	176	3.551	0.943	0.071			
	SE	168	3.601	0.913	0.070			
	EI	101	3.475	1.046	0.104			

**Table 11:** Factors affecting undergraduate students’ sustain use intention by university.

Factor	University	N	Mean	SD	SE	df	F	P
RA	Private university	429	3.486	0.890	0.043	1	0.079	0.779
	Public university	374	3.468	0.895	0.046			
CP	Private university	429	3.448	0.904	0.044	1	0.521	0.469
	Public university	374	3.493	0.870	0.045			
TL	Private university	429	3.419	0.912	0.044	1	2.576	0.108
	Public university	374	3.520	0.866	0.045			
OS	Private university	429	3.466	0.972	0.047	1	1.283	0.257
	Public university	374	3.543	0.937	0.048			
PU	Private university	429	3.449	0.895	0.043	1	0.899	0.343
	Public university	374	3.508	0.879	0.045			
PE	Private university	429	3.448	0.902	0.044	1	0.347	0.555
	Public university	374	3.485	0.872	0.045			
PA	Private university	429	3.409	0.935	0.045	1	2.203	0.137
	Public university	374	3.505	0.891	0.046			
SISU	Private university	429	3.447	1.000	0.048	1	3.215	0.072
	Public university	374	3.570	0.945	0.049			

**Table 12:** Factors affecting undergraduate students’ sustain use intention by grade.

Factor	Grade(semesters)	N	Mean	SD	SE	df	F	p
RA	1–2	195	3.557	0.861	0.062	3	1.405	0.240
	3–4	226	3.487	0.902	0.060			
	5–6	198	3.487	0.894	0.064			
	7–8	184	3.371	0.907	0.067			
CP	1–2	195	3.498	0.873	0.063	3	0.778	0.492
	3–4	226	3.412	0.885	0.059			
	5–6	198	3.532	0.847	0.060			
	7–8	184	3.441	0.951	0.070			
TL	1–2	195	3.539	0.852	0.061	3	1.338	0.260
	3–4	226	3.407	0.906	0.060			
	5–6	198	3.521	0.878	0.062			
	7–8	184	3.401	0.926	0.068			
OS	1–2	195	3.615	0.897	0.064	3	2.273	0.079
	3–4	226	3.445	0.972	0.065			
	5–6	198	3.559	0.932	0.066			
	7–8	184	3.390	1.011	0.075			
PU	1–2	195	3.556	0.837	0.060	3	0.919	0.415
	3–4	226	3.488	0.892	0.059			
	5–6	198	3.439	0.891	0.063			
	7–8	184	3.417	0.929	0.069			
PE	1–2	195	3.470	0.849	0.061	3	0.014	0.997
	3–4	226	3.471	0.862	0.057			
	5–6	198	3.455	0.855	0.061			
	7–8	184	3.465	0.995	0.073			
PA	1–2	195	3.488	0.912	0.065	3	0.516	0.680
	3–4	226	3.436	0.923	0.061			
	5–6	198	3.496	0.862	0.061			
	7–8	184	3.395	0.967	0.071			
SISU	1–2	195	3.520	0.995	0.071	3	0.437	0.727
	3–4	226	3.535	0.963	0.064			
	5–6	198	3.520	0.974	0.069			
	7–8	184	3.433	0.979	0.072			

## Discussion

Many scholars have conducted extensive research on students’ willingness to sustain using, especially the research on the combination of information technology and education. Researchers use PLS-SEM to analyze influencing factors in undergraduate students’ sustained use of open source communities. Using a mixed model (IDT-TAM), the researchers found that RA, CP, PE, TL, OS, PU, and PA were key determinants of sustained usage intention among undergraduate students. Based on the research results of model reliability and validity, we can see that the model is suitable for this study. All paths in the model are positively and significantly correlated.

The research purposes of this study were to identify the factors affecting undergraduate students’ sustained usage of open source communities. The interrelationships among the factors are mainly used to solve research questions 1–3 of this study. For research question 1. The research results show that RA, CP, PE, and TL all have a significant positive impact on PA. The research results support the hypotheses H_1.1a_, H_1.1b_, H_1.1c_, and H_1.1d_. This result is contrary to the findings of some researchers [62–64]. Their results appeared because the studies came from different countries and there was a localization phenomenon. In previous studies, Al-Rahmi et al. (2019), Shiau et al. (2018), Almaiah et al. (2022) and Huang et al. (2020) found that RA, CP, PE, TL is the decisive factor of PA, which is consistent with the results of this study [17–19, 65]. It shows that the high level of relative advantage (RA), compatibility (CP), perceived ease of use (PE), and trialability (TL) have a high level of positive impact on undergraduate students’ perceived attitudes. In order to increase students’ attitudes toward sustained use of open source communities, undergraduate students’ positivity regarding relative advantage, compatibility, perceived ease of use, and trialability should be increased. In addition, the results show that PE, TL, and OS have a significant positive impact on PU, and the research results support the hypotheses H_1.2a_, H_1.2b_, and H_1.2c_. This result is contrary to the findings of some researchers [66, 67]. This may be due to the differences in the user groups they studied. The results of this study are consistent with the findings of some researchers [17–19, 65]. The research results also show that positive PE, TL, and OS can have a positive impact on PU.

In addition, for research question 2. Research data shows that PA and PU have a significant positive impact on SISU. In the IDT-TAM model, PA and PU have positive and significant mediating effects. This result supports the research hypotheses H_2.1a_ and H_2.1b_. This result is contrary to the findings of some researchers [68, 69]. This is due to the different prior experiences of the users they studied. In other words, RA, CP, PE, TL, and OS can have a significant impact on SISU through PA and PU. The results of Li et al. (2022), Qin et al. (2022), and Wang et al. (2021) are consistent with the results of this study [40, 41, 46].

In addition, for research question 3. The research results also confirmed that PU can have a positive and significant impact on PA. The results confirmed the research hypothesis H_3_. The above hypothetical path is shown in Fig 1. This result is contrary to the findings of some researchers [70]. The findings of Li et al. (2022), Wang et al. (2021), and Liu (2017) are consistent with those of this study [40–42]. Explain that high perceived usefulness can increase perceived attitude positivity. Simultaneously, it also proves the significance of PU in PA. Universities and teachers can encourage the trial of open source communities and foster the sharing of experiences among students to enhance the perceived usefulness of open source communities among undergraduate students, thus improving their attitudes.

Regarding research question 4 of this study, the primary focus of this research model is to examine variations in factors influencing undergraduate students’ sustained use of open source communities based on gender, major, university, and grade. Findings showed no significant differences in terms of major, gender, university, and grade based on the model. In the theory of innovation diffusion, the results of this study are contrary to those of Liu (2019), Richardson (2009), and Du (2019) [22, 38, 39]. In the technology acceptance model, the findings are opposite to those of Nyamekye et al. (2022) [50], but consistent with those of Wang & Houdyshell (2021) [51]. Obviously, the IDT-TAM model effectively predicts the factors that affect undergraduate students’ willingness to sustain using open source communities. Therefore, we can increase the enthusiasm of undergraduate students for RA, CP, PE, TL, OS, PA, and PU, thereby increasing undergraduate students’ willingness to sustain using open source communities. Open source talents can be better cultivated and trained when undergraduate students are willing to sustain using open source communities for learning.

According to the above results, it can be seen that in terms of relative advantage. Undergraduates believe that open source communities have learning advantages, which play an important supporting role in undergraduates’ perceived attitudes. In order to promote the cultivation of open source talents, colleges, and universities should promote the benefits of open source community platforms, let students experience the advantages of open source platforms through practical operations, and increase undergraduate students’ positive attitude towards open source communities. Also, in terms of compatibility. Undergraduate students believe that learning in open source communities can be beneficial and compatible with their previous experiences, thus playing a key role in undergraduate students’ perceived attitudes. Universities should pay attention to the compatibility of open source software to improve undergraduate students’ attitudes toward the open source community.

Furthermore, in terms of perceived ease of use. Undergraduate students believe that using open source communities for learning is easy for them, emphasizing the need for undergraduate students in terms of their perceived attitudes and perceived usefulness. Therefore, teachers should pay attention to the ease of use of open source communities in their teaching design, and before using open source projects, relevant knowledge is taught to increase the ease of practical tasks and mobilize undergraduates’ positive attitudes towards the open source community. At the same time, undergraduate students are prompted to view open source communities as more useful. In addition, in terms of trialability. Undergraduate students believe that open source communities are free and can be tried without time limits, which increases positivity in terms of perceived attitudes and perceived usefulness. Universities and teachers should encourage undergraduate students to try open source communities and can also formulate relevant incentive policies to promote undergraduate students’ perceived attitudes and perceived usefulness towards open source communities. Also, in terms of observability. Undergraduate students can easily observe others using open source communities for learning, thus playing a decisive role in perceived attitudes and perceived usefulness. Colleges and universities should encourage undergraduate students to share their learning experiences in open source communities, so that more students can observe the benefits of open source communities, thereby promoting the positivity of perceived attitudes and perceived usefulness.

In terms of perceived attitudes. Undergraduate students have a positive attitude towards the open source community, which plays a key role in undergraduate students’ sustained use intention. Furthermore, in terms of perceived usefulness. Undergraduate students believe that learning in the open source community can be useful for their own learning, thus playing an important role in undergraduate students’ sustained use intentions. Emphasizing the need for perceived attitudes and perceived usefulness. Universities and teachers can increase PA and PU by increasing undergraduate students’ RA, CP, PE, TL, and OS to the open source community, thereby enhancing undergraduate students’ willingness to sustain using them. Universities can increase undergraduate students’ attitudes towards open source communities and their perceived usefulness by focusing on aspects such as promoting open source communities, learning about students’ previous experiences, and improving their compatibility with the open source community, highlighting the advantages of open source community platforms, free unlimited trials, sharing user experiences, and teaching students relevant knowledge before using open source projects. At the same time, it will boost undergraduate students’ sustained willingness to use open source communities.

### Implication

#### Theoretical implication

The results of this study have two implications. The theoretical impact is that this study is based on the IDT-TAM mixed model and is formed by replacing complexity with PE. In addition, based on the model, this study also considers the influence of control variables on factors in the model. This study validates the IDT-TAM model and increases universities’ understanding of open source talent training. Such research adds to the literature on universities’ willingness to sustain using open source communities for open source talents. Although the revision of this research model is not large, there are very few studies on open source talents’ willingness to sustain using open source communities in China. Therefore, this study fills the gap in this area and provides inspiration for universities to better cultivate open source talents.

#### Practical implication

Secondly, in terms of implementation significance, important suggestions are put forward for teachers, open source community platforms, policy makers in colleges and universities, and school administrators. According to the research results, students’ perceived attitudes and perceived usefulness can be improved by increasing students’ enthusiasm for the open source community’s relative advantages, compatibility, perceived ease of use, trialability, and observability. And on the basis of achieving high levels of perceived attitude and perceived usefulness, students’ willingness to sustain using the open source community will be enhanced. Such as teachers can design courses and projects based on open source technologies that encourage student autonomy and motivation. The open source community can improve the functional system of the open source community and improve the usefulness of learning in the open source community. For open source community managers, they should understand the demand and current situation of open source talents and organize more community activities to attract and maintain the continuity of users. Policymakers at institutions of higher learning should establish and improve appropriate guidelines or policies regarding open source learning for students. For university administrators, students should be supported in learning in open source communities. Developers can actively participate in open source projects and community activities, find interests, and improve skills to sustain contributing to the open source community.

## Conclusions

This study mainly refers to the IDT-TAM mixed model, this model is utilized to analyze the factors influencing undergraduate students’ sustained use of open source communities. The research purposes are to identify factors that contribute to the development of open source talents in a context where such talents are scarce. Because universities are important contributors to the cultivation of open source talents. Hence, by identifying the factors influencing open source talents’ intention to sustain in using open source technology can we better mobilize the enthusiasm of undergraduate students and maintain long-term and voluntary learning and practice. In order to cultivate high-quality open source talents.

Regarding research question 1, the results of this study show that RA, CP, PE, TL, and OS promote PA and PU. It is proved that there is a positive relationship between IDT and TAM. In addition, regarding research question 2, the research results confirmed that both factors, PA and PU have a positive effect on SISU. It shows that the factors in IDT-TAM can positively affect SISU open source communities. In addition, in response to research question 3, the research results confirmed that the PU of the TAM model can positively affect PA. Finally, for research question 4, there are no differences among TAM, IDT, and SISU at different levels of demographic information, namely gender, major, university, and grade.

### Limitations and future suggestions

While the study’s theory provides new insights into the research, there are some limitations. Using only data from China when studying theoretical and practical issues affects undergraduate students’ willingness to sustain using open source communities. Hence, future research suggestions include data from other countries. In addition, the sample size used for data analysis in this study was 803, which is limited. It is recommended that the sample size be increased in future research. In addition, this study only considered the factors in the IDT-TAM model when using quantitative research methods, so the analysis has certain limitations. Therefore, follow-up research can fill this research gap by analyzing other regions and considering other influencing factors.

## Supporting information

S1 File Questionnaire. https://doi.org/10.6084/m9.figshare.26466961.
(PDF)

S1 Data https://doi.org/10.6084/m9.figshare.25330651.v1.
(XLSX)
