# Supplementary material for: Investigating the factors that influence Chinese undergraduate students’ sustained use of open source communities
Source: PLoS One. 2024 Dec 27;19(12):e0308005. doi: 10.1371/journal.pone.0308005 (PMC11676490; doi:10.1371/journal.pone.0308005)
Supplement: S1 File — https://doi.org/10.6084/m9.figshare.26466961. (PDF) [file pone.0308005.s001.pdf]

A questionnaire on the factors influencing the willingness of undergraduate students in Yunnan, China to sustain using open source communities in 2023

Demographic information

1. Gender
a. Female
b. Male

2. Age range
a.18-19
b.20-21
c.>22

3. Major
a. Computer Science and Technology
b. Electronic Information Science and Technology
c. Software engineering
d. Electrical information

4. Grade（Semester）
a.1-2
b.3-4
c.5-6
d.7-8

5. What is the nature of your school?
a. Private;
b. Public;

6. Have you ever used an open-source community (Gitee University version) for learning?
a. Yes
b. No

7. How often do you use the open-source community for learning?
a. Several times a week
b. Once a week
c. Once every two weeks
d. Once a month
e. Once every six months or more

8. When was the last time you used an open-source community for learning?
a. Within a week
b. Within half a month
c. Within a month
d. Within a year
e. More than a year ago
f. Never

9. Are you currently skilled in the use of open-source community rules?
a. Very skilled
b. Relatively skilled
c. Average
d. Not very skilled
e. Not at all skilled

10. Who has helped you the most in learning to use the open-source community proficiently?
a. Teacher
b. Classmates
c. Friend
d. Parents
e. Other

In the following table, 1 represents Strongly Disagree, 2 represents Disagree, 3 Neither Disagree Nor Agree, 4 represents Agree, and 5 represents Strongly Agree.

A. Innovation diffusion theory (IDT)

NO	Statement	1	2	3	4	5
11	Using open-source communities for learning enables me to complete tasks more quickly.					
12	Using open-source communities has improved the quality of my learning.					
13	Using open-source communities has made my learning easier.					
14	Using open-source communities has increased my learning efficiency.					
15	Using open-source communities allows me to better control my learning progress.					
16	Overall, I find that using open-source communities is beneficial to my learning.					

Measurement Scale References (Stark, 2018).

NO	Statement	1	2	3	4	5
17	Using open-source communities is compatible with most aspects of my learning.					
18	I can quickly adapt to the learning methods of open-source communities.					
19	Using open-source communities for learning is suitable for my learning style.					
20	The technical support provided in the open-source community learning environment is compatible with my learning habits.					
21	Many of the programs I use in open-source communities are compatible with other learning methods.					
22	I have the skills necessary to use open-source communities for learning.					

Measurement Scale References (Meng, 2021).

NO	Statement	1	2	3	4	5
23	I think it's easy to try out the open-source communities.					
24	I know where I can go to satisfactorily try the various features of open-source communities.					
25	I was permitted to use open-source communities on a trial basis long enough.					
26	I am able to experiment with open-source communities as necessary.					
27	The open-source community is open enough for me to test the various features it provides.					

Measurement Scale References (Dotter, 2018).

NO	Statement	1	2	3	4	5
28	I have no trouble explaining why using open-source communities is beneficial.					
29	The benefits of using open-source communities are obvious.					
30	I can easily see how others have benefited from learning through the use of open-source communities.					

Measurement Scale References (Pinho et al., 2021)

B. Technology Acceptance Model (TAM)

NO	Statement	1	2	3	4	5
31	Using open-source communities helps me better collect learning resources and information.					
32	Using open-source communities helps me better understand learning content.					
33	Using open-source communities helps me choose the necessary resources according to different needs.					
34	Using open-source communities facilitates better interaction with classmates.					
35	Overall, the use of open-source communities is very helpful to my learning.					

Measurement Scale References (Zhang et al., 2015)

NO	Statement	1	2	3	4	5
36	I can easily collect the learning resources I need in open-source communities.					
37	I can easily present my learning results effectively through open-source communities.					
38	In the open-source community, I can easily choose different learning resources according to different needs.					
39	During the use of open-source communities, I can achieve effective interaction at any time.					
40	Overall, the use of open-source communities is easy for me.					

Measurement Scale References (Zhang et al., 2015)

NO	Statement	1	2	3	4	5
41	I am glad that I can now learn through the open-source community.					
42	I feel happy when learning through open-source communities.					
43	Open-source communities allow me to quickly obtain knowledge.					
44	I think that using open-source communities can save me time.					
45	Open-source communities will motivate me to do more learning.					

Measurement Scale References (Ab Jalil et al., 2019)

C. The Students' intention of Sustained usage (SISU)

NO	Statement	1	2	3	4	5
46	I am willing to continue using open-source community platforms frequently for learning.					
47	In the future, I will use open-source community platforms frequently for learning.					
48	I am willing to recommend the open-source community platform I have used to others."					

Measurement Scale References (Li, 2016)
